# Universal Autism Screening in Early Learning Programs: A Feasibility Study

**DOI:** 10.3390/children13060775

**Published:** 2026-06-02

**Authors:** Thyde Dumont-Mathieu, Marianne Barton, Rosalie Chuckta, Natalia Suarez Martinez, Deborah Fein

**Affiliations:** 1 Division of Developmental-Behavioral Pediatrics, Connecticut Children’s, Hartford, CT 06106, USA; 2 Department of Pediatrics, University of Connecticut School of Medicine, Farmington, CT 06030, USA; 3 Department of Psychological Sciences, University of Connecticut, Storrs, CT 06269, USA; 4 Department of Research, Connecticut Children’s, Hartford, CT 06106, USA

**Keywords:** universal screening, early learning program, autism spectrum disorder, developmental conditions, early intervention

## Abstract

**Highlights:**

**What are the main findings?**

- Autism-specific screening occurred with 99% of families who were approached to participate in screening within their early learning program setting. Ninety three percent (93%) of families whose children screened positive followed through with recommended next steps.
- One hundred percent (100%) of those evaluated were eligible for early intervention services, and 31% received an autism educational classification through early intervention.

**What are the implications of the main findings?**

- Autism-specific screening can be feasibly incorporated into the workflow of an early learning program.
- Screening and early intervention referral can be completed with high fidelity by trained early learning program staff.

**Abstract:**

**Background**: The American Academy of Pediatrics recommends autism-specific screening at the 18- and 24-month well-child-care visits. Early identification facilitates early intervention (EI), which improves developmental outcomes. Historically, Non-Hispanic Black and Hispanic/Latino children in the United States receive autism diagnoses and autism-specific services later than Non-Hispanic White children. Variability in pediatric screening rates may indicate that systemic factors impede screening and referral; enhanced screening across community settings may support autism identification and connection to services. **Methods**: A feasibility study was conducted with one early learning program (ELP) to determine if screening for autism in ELPs is feasible. ELP teachers and staff received one 90 min training session on screening with the Modified Checklist for Autism in Toddlers—Revised (M-CHAT-R). They were then tasked with independently screening ELP-enrolled children between 16 and 30 months old. **Results**: Eighty children were eligible for screening and 79 screenings were completed; 14 screens were positive and 65 were negative. Of the 14 positive screens, eight referrals were made to EI. All eight families completed EI evaluations and were eligible for EI services. One family declined an evaluation. Five positive autism screens were for children already receiving general EI services. Those five screening results were communicated to the child’s EI team and an autism-specific evaluation was completed; four of the five children subsequently received autism diagnoses. **Conclusions**: Our data supports the feasibility of completing autism-specific screenings within an ELP setting.

## 1. Introduction

Early intervention (EI) is underutilized in the treatment of neurodevelopmental conditions, including autism spectrum disorders (hereafter referred to as autism) [1,2]. All states are required to have an EI program due to the Individuals with Disabilities Act (IDEA) Part C Program; therefore, EI has the potential to serve all children under three years old with developmental difficulties and their families. In Connecticut, both an EI eligibility evaluation and autism evaluation are completed for children who have an elevated likelihood of autism based on parent concern, provider concern, or positive autism screening. Children may qualify for EI services due to general developmental concerns, autism concerns, or both [3]. Autism evaluations completed by EI lead to an educational classification of autism. Educational eligibility is decided by the EI team (e.g., licensed clinical social workers) and eligibility qualifies children for autism-specific services under IDEA. This is an important distinction from a medical diagnosis of autism, which is made by a specialty trained physician (e.g., Developmental–Behavioral Pediatrician) or clinician (e.g., Licensed Clinical Psychologist) [4,5]. Autism affects as many as 1 in 31 children [6]. Characteristics typically present before the age of three [7] and autism diagnoses have been found to be stable for patients as young as 14 months old [8]. Identifying children with an elevated likelihood of autism and connecting them to EI as early as possible improves autism-related and overall child outcomes [9,10,11].

Historically, there have been racial/ethnic disparities in the age of autism diagnosis and start of appropriate EI services [12,13,14,15,16,17,18]. As a result of these delays, many children miss the opportunity to receive appropriate EI services and the associated improvements in developmental outcomes [13,19,20].

Since 2006, the American Academy of Pediatrics has recommended that pediatricians and pediatric clinicians augment developmental surveillance of children during well-child-care (WCC) visits with universal screening for general developmental conditions and autism [21]. Autism screening with validated tools, such as the Modified Checklist for Autism in Toddlers, Revised (M-CHAT-R) [22], is recommended at the 18- and 24-month WCC visits.

Despite this recommendation, more than one-third of all children are not being formally screened during their 18- and 24-month WCC visits, and rates of screening, diagnosis, and connection to EI services are lower among Black and Hispanic/Latino children (hereafter referred to as children of color) [23,24,25,26]. A lack of screening and connection to services for more than one-third of eligible children is concerning, since early access to EI services leads to improved developmental outcomes in children which last at least two years following services [23,27,28,29,30,31,32]. Universal screening can improve detection rates and referral to services for children of all racial and ethnic backgrounds and has been shown to reduce or eliminate racial and ethnic disparities in autism identification [1,2,15,23,33,34,35,36].

Additional opportunities to screen and refer children with an elevated likelihood of autism outside of the pediatric office may improve screening rates. One option is to screen children in a setting that they regularly attend, such as early learning programs (ELP), which may eliminate logistical barriers to autism-specific screening, evaluation, and connection to services.

ELPs include Early Head Start (EHS) and programs in community-embedded organizations such as the YWCA and YMCA. EHS eligibility criteria requires that all children enrolled in EHS programs are either from families with incomes below the poverty guidelines, from families who are facing homelessness, foster children (regardless of their foster family’s income), or from families receiving public assistance [37]. In 2023–2024, EHS programs served approximately 200,000 American children aged 0–5; 77% of these children were children of color [38]. Additionally, there are nearly 200 YWCAs and almost 2600 YMCA programs that serve young children in the United States [39,40]. Although EHS programs require general developmental screening, they do not routinely conduct autism-specific screening.

ELPs are a promising setting to supplement pediatric autism screenings. By leveraging existing parent–teacher relationships, families may be more likely to acknowledge developmental concerns and follow through with EI referrals [41]. Families interact with ELP teachers far more frequently than pediatricians or pediatric clinicians: at 30 months old, a child typically has had 11 WCC visits, whereas that same child would have 11 encounters with their ELP teacher in approximately two weeks [42]. ELP teachers are variably trained in child development, engage with children in the context of same-aged peers, and have trusted relationships with families [43].

To date, there have been two published studies on autism screening involving ELP teachers. In a United States-based study, first-authored by Janvier [44], childcare teachers screened 967 children in Head Start and non-Head Start childcare centers in New Jersey using the Social Communication Questionnaire [45], the M-CHAT [46], or both. The mean age at screening was 50 months old, well past the age served by EI; in that study, only 40 children were screened with the M-CHAT at 16–30 months old [44]. The Janvier study demonstrated that recruiting families of children who are primarily 3 years old and older to participate in screening from childcare centers (88% of families consented to participate) and conducting teacher-completed screening (screening occurred with 90% of the families who consented) is feasible [44]. These results are promising in our study’s aim of assessing the feasibility of facilitating parent- and teacher-completed autism-specific screening within ELPs. Another international study done in ELPs was limited because it focused on instrument development and, as such, did not use a validated measure [47].

The aim of this study was to train ELP staff to conduct autism-specific screening of children 16–30 months old within a single ELP site in Connecticut. We sought to determine the feasibility of completing parent and teacher autism-specific screeners within the ELP center, and whether existing teacher–parent relationships facilitated connecting families to EI services for positive screens.

## 2. Materials and Methods

**Figure 1:**
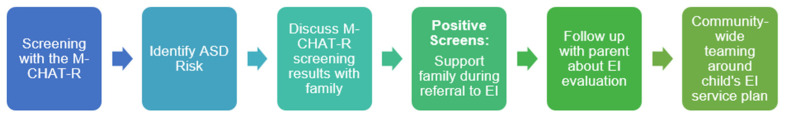
Bridging the Gap model.

The Bridging the Gap model was developed by the first author (Figure 1) with a goal to train ELP teachers and other relevant ELP staff (e.g., social workers, mental health managers, disability specialists/coordinators) to conduct autism screenings within ELPs, communicating results to families in their preferred language (English or Spanish), and maintain active cross-system collaboration between ELPs and EI programs from screening to EI connection.

This study was conducted in partnership with one ELP in Connecticut that had a large racially/ethnically diverse student enrollment. Screening occurred between September 2019 and November 2022. The study was conducted both before and during the COVID-19 pandemic.

Study procedures were approved by our institution’s Scientific Review Committee and Institutional Review Board (protocol #17-136).

An ELP serving a high proportion of children of color was identified and recruited. The ELP is an EHS-designated site, and 100% of children enrolled were included in the EHS program. Upon site enrollment, 100% of ELP staff agreed to participate. ELP staff participated in one 90 min focus group and one 90 min training session with the first author.

A senior staff member at the site was designated as the facilitator for the study; the facilitator knew all enrolled families in the program, and families knew the facilitator. The facilitator was a master’s-level Social Worker with over 20 years of experience as a Mental Health and Disabilities Manager. As a Mental Health and Disabilities Manager, the facilitator’s usual role was to coordinate special needs services for children and families, support families in developmental screenings, and connect children and families to EI and other resources (such as special education for children over three years old). Site and community demographics were provided by the ELP facilitator.

Eighty children between the ages of 16–30 months were eligible to participate. Consistent with state mandates, each classroom had 1:4 ratio for classroom teacher to children aged 16–30 months old. Each classroom had eight children and two teachers. As an EHS-designated ELP, this site required a minimum of two parent conferences and two home visits per year. Parent education sessions on various topics (i.e., community resources) were routinely offered by the ELP. All ELP staff were required to complete a minimum of 15 h of professional development training per year.

The study team collected information on the program’s enrollment, staffing, unmet program needs, communities served, current developmental screening practices, and current EI referral processes. As an EHS-designated ELP, general developmental screening was required to be completed 45 days post child enrollment. This ELP used the Ages and Stages Questionnaire, Third Edition (ASQ-3) [48], for developmental screening and completed it for all children 45 days post child enrollment. Screening was repeated three times per year. As the Mental Health and Disabilities Manager, the ELP facilitator’s role was to support EI referrals if developmental concerns were noted (either through a positive ASQ-3 screening, parent concern, or classroom teacher’s observation). The top unmet program needs reported by ELP staff were consistent staffing, nutritional resources for families, and additional ways to collaborate within the community.

Once the ELP was enrolled, the first author led staff onboarding, beginning with a focus group with 16 ELP staff members, including classroom teachers, the Mental Health and Disabilities Manager, EHS coordinators, family advocates, nurse consultants, and home visitors. The focus group explored staff members’ perceived barriers to early identification of children with developmental–behavioral difficulties and their connection to EI services (qualitative findings to be published separately).

After the focus group, ELP staff members participated in one 90 min training session with the first author to discuss developmental milestones, differences between developmental screening and diagnostic evaluation, an overview of developmental conditions and autism, an overview of EI including how to refer to EI, administration and scoring of the M-CHAT-R, available resources, and project logistics (including verbally consenting parents/guardians [hereafter referred to as parents]). Pre- and post-tests developed by the study team were completed at the beginning and end of the training. Pre- and post-tests consisted of 10 true/false questions on topics including child development, identifying concerns, screening versus evaluation, autism in general, and EI. The first author completed this 90 min training session with all teachers and ELP staff currently working within the ELP at the start of the study, and over the multi-year study, the first author repeated this training with newly onboarded ELP staff to ensure that all ELP staff received the same training.

Two weeks after training completion, the study research assistant (RA) facilitated a “screening kick-off” meeting with the ELP facilitator to review study procedures and data collection protocols, including verbally consenting parents, M-CHAT-R scoring, and referring to EI.

The ELP facilitator was responsible for (1) confirming the number of eligible children enrolled in the program; (2) verbally consenting each interested parent for enrollment; (3) providing the M-CHAT-R to the parent of each enrolled child for completion and collecting the parent-completed screener; (4) providing the M-CHAT-R to each classroom teacher for completion and collecting the teacher-completed screener; (5) scoring the parent and teacher M-CHAT-R screenings; (6) scheduling a meeting with each family to discuss screening results; and (7) assisting with EI referrals when indicated.

Eligible children were screened through completion of the M-CHAT-R [22], a validated 20-item (yes/no) screening checklist available for free in multiple languages including Spanish, by both the child’s parent and classroom teacher. In this study, families completed the M-CHAT in either English or Spanish. If the parent screen, teacher screen, or both screens were positive, the screen was considered positive and the facilitator offered to assist the family in a referral to EI for evaluations (eligibility and autism-specific). As per the M-CHAT-R scoring guidelines, screens are considered positive if more than two responses indicate an increased likelihood for autism. If the child was already receiving general EI services, the facilitator helped the parent connect with the child’s EI team to communicate the positive M-CHAT-R screening results; those children were then eligible for an EI autism evaluation.

ELP teachers were responsible for completing M-CHAT-R screenings for each child enrolled in the study based on their classroom observations of the child.

Enrolled parents were responsible for completing an M-CHAT-R and meeting with the ELP facilitator to discuss screening results.

Fidelity was assessed through 15–30 min bi-weekly to monthly meetings between the RA and ELP facilitator. Meetings occurred in person, via Zoom or over the phone. During the COVID-19 pandemic, fidelity meetings only occurred via Zoom or phone. Fidelity check-in meetings reviewed the project protocol and verified how many children were eligible for screening, how many families consented/declined to participate in the project, parent and teacher screening results, confirmation that discussions about screening results occurred, and how many families chose to follow through with an EI referral or declined. Fidelity checks also verified all data collected regarding each child and family and available EI referral outcomes. Fidelity meetings demonstrated that 100% of the ELP team (facilitator and classroom teachers) followed the protocol with high fidelity and rigor. The Fidelity Check-in Guide (Table 1) shows the themes of this structured fidelity check-in.

As this study occurred before and during the COVID-19 pandemic, study procedures only occurred when the ELP site was open. During intermittent site closures at the peaks of the COVID-19 outbreak, no screening occurred, but screening resumed once the site was reopened.

## 3. Results

Twenty-four ELP staff (classroom teachers, Mental Health and Disabilities Manager, EHS coordinators, family advocates, nurse consultants, and home visitors) participated in one 90 min training session with the first author. After training completion, mean pre- and post-test scores indicated gains in learning (pre-test scores = 86.5% vs. post-test scores = 93.5%). A total of 80 families were eligible to participate in the project. One family declined participation and 79 (99%) parents were verbally consented by the ELP site facilitator; all 79 children (100%) were screened with the M-CHAT-R by both their parent and their classroom teacher.

**Figure 2:**
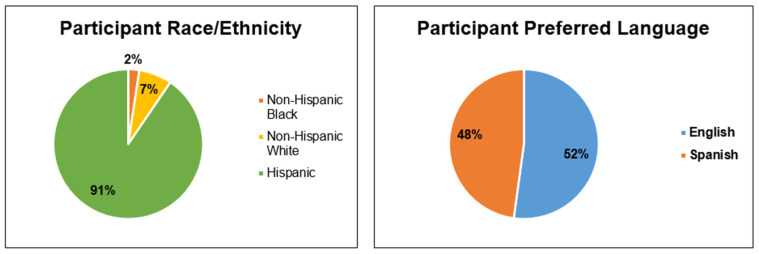
Participant demographics.

In the first year of screening, only screening completion (yes/no), results of screening (positive/negative), and outcomes of screening and EI evaluation if applicable (EI referral/EI evaluation/EI eligibility) were collected. After the first year of screening, the study collected participant-specific data such as race/ethnicity and preferred language. Race/ethnicity and language preference data was collected for 43 enrolled children (54% of our sample). Of the race and ethnicity data available, 91% identified as Hispanic/Latino, 2% as Non-Hispanic Black, and 7% as Non-Hispanic White. Preferred language data were available for 87% of the enrolled children; 52% reported English as their preferred language and 48% reported Spanish as their preferred language. Families who reported Spanish as their preferred language completed the M-CHAT-R in Spanish, and families who reported their preferred language as English completed the M-CHAT-R in English. Teachers completed the M-CHAT-R in English for 61% of the children and in Spanish for 39% (Figure 2).

Of the 79 completed M-CHAT-R screens, 14 were positive and 65 were negative (18% positivity rate). Of the 14 positive screens, 11 screens were positive on both the parent- and teacher-completed M-CHAT-R screens, and three screens were positive on only the teacher-completed M-CHAT-R screen. On average, teachers knew the child for whom they were completing the M-CHAT-R for 2.44 months (range: 1.5–12 months). While there was agreement for 79% of the positive screens, of the three children who screened positive on the teacher screener only, two of those families completed an EI evaluation and qualified for EI services, highlighting the teacher-completed screening’s value in identifying children who may benefit from EI services.

**Figure 3:**
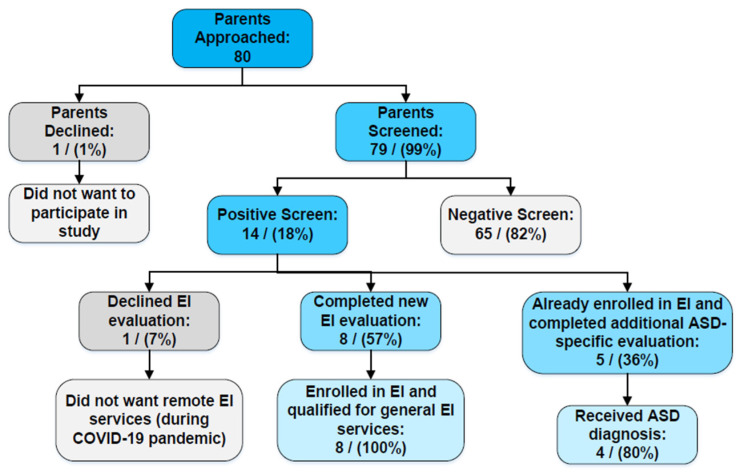
Screening outcomes.

Of the 14 positive screens, 13 referrals to EI were completed (93%) (Figure 3). One family (parent-negative screen/teacher-positive screen) declined an EI referral because they did not want remote EI services during the COVID-19 pandemic.

The facilitator assisted eight of the 13 families (57%) with a new EI referral; these eight families completed both an EI eligibility evaluation and autism-specific EI evaluation for their child. All eight children (100%) qualified for general EI services (i.e., developmental delay, speech delay) and none received an autism educational classification.

Five of the 13 families (36%) who screened positive on the M-CHAT-R were already enrolled in EI for general services. After screening positive on the M-CHAT-R as part of their study participation, screening results were communicated to the child’s EI team and all five families completed an autism-specific EI evaluation; four of the five children (80%) then received an autism educational classification.

As screening occurred before and during the COVID-19 pandemic, of the 79 screens completed, 37 (47%) were completed pre-COVID-19 pandemic. Five of these screens were positive. Three of the five positive screens were for children already enrolled in EI. Those three children completed an autism-specific EI evaluation and received an autism educational classification. The other two screens completed EI evaluations and those children qualified for EI due to general developmental delays.

During the COVID-19 pandemic, 42 (53%) screens were completed; nine were positive and eight went through with an EI referral. Two children were already enrolled in EI services. Those children completed an autism-specific EI evaluation and one received an autism educational classification. One family declined an EI referral for their child due to not wanting remote EI services, a pandemic-specific reason for declining an EI evaluation. The remaining five children all completed EI evaluations and qualified for EI due to general developmental delays.

Of the 36 screens completed in English, six were positive; of the 33 screens completed in Spanish, eight were positive. The one family who declined an EI referral during the COVID-19 pandemic indicated Spanish as their preferred language.

## 4. Discussion

In total, 99% of families approached by the ELP facilitator consented to participate in the study, and 100% of those who consented followed through with M-CHAT-R screening completion. Ninety three percent (93%) of families whose child screened positive (either on the parent screening, teacher screening, or both) followed through with either a new EI referral or contacted their existing EI provider to complete additional autism evaluations. Eight of the 13 families who screened positive on the M-CHAT-R completed a new EI eligibility evaluation and autism-specific EI evaluation. One-hundred percent (100%) of these families were eligible for general EI services, and none received an autism educational classification. Of the five families who were already receiving general EI services, all five completed an additional autism EI evaluation and four (80%) received an autism educational classification.

Data collected during year two of the study period demonstrate our project’s ability to recruit racially/ethnically diverse families (93% families of color for the data available).

M-CHAT-R positive screening rates vary widely across the literature from 3% to 20% [22,23,34,46,49,50,51,52,53,54]; our positive screen rate of 18% falls within this range. Our study demonstrates that autism screening can be done in a busy ELP setting, and has the ability to reach a large number of children and families.

Of note, four of the thirteen (31%) children who screened positive with the M-CHAT-R and completed an EI evaluation received an autism educational classification and nine (69%) received general developmental delay diagnoses by EI. The M-CHAT-R validation study also reported on the M-CHAT-R’s ability to identify general developmental delays, as well as autism [22]. The validation paper noted that although one might interpret this to mean that the M-CHAT-R can be used more broadly than for autism, such use would not be justified given that the sensitivity for using the M-CHAT-R to detect non-autism delays is not known [22].

Our study also demonstrates that screening and EI referral can be completed in an ELP setting with high fidelity. Fidelity meetings between the RA and ELP facilitator confirmed screening can be completed within the ELP’s existing workflow, and that ELP teachers and other staff support the goals of screening as a means of identifying children with developmental challenges earlier and connecting them to EI services. By embedding autism screening procedures within existing developmental screening protocols, screening within ELPs was sustainable even during the COVID-19 pandemic. Therefore, ELPs are a promising venue for augmenting the autism screening and EI referral currently conducted by pediatricians and pediatric clinicians as part of WCC visits. By leveraging existing teacher–parent trusting relationships, ELPs have the potential to be a valuable addition to the current screening practices and, therefore, provide an opportunity to reach additional children in under-resourced communities [44]. This study’s high participation rate and high rate of following through with EI referral and evaluation are unique due to the presence of a dedicated and experienced facilitator. Additionally, three families screened positive on teacher-completed M-CHAT-R screens only. As might be expected, these families did not identify the same concerns for their child on the parent-completed screener; however, discussion about the positive results with their trusted facilitator facilitated the completion of EI referral, evaluation and start of EI services for two families, highlighting the potential impact of multi-informant screening by caregivers who know the child’s development [55,56], the value of teacher input as parents and teachers observe the child in different contexts [56], and overall parent–teacher trust [57,58]. Teachers in particular have the benefit of observing children with same-aged peers engaging in a structured setting. Additionally, through their training and/or experience [43], teachers may have more child development knowledge than some parents.

Through focus groups, ELP staff, facilitators, administrators, and classroom teachers enthusiastically supported the goals of screening for autism and expediting an EI referral for children with an elevated likelihood of autism to connect them to diagnostic and therapeutic services faster. ELP staff helped to streamline frequent re-trainings and project onboarding for newly hired classroom teachers. Our results support the possibility that screening within ELPs is an effective adjunct to autism screening conducted at primary care pediatric sites.

Given the success of the feasibility study, the study team has expanded autism screening to other ELPs and have incorporated lessons learned into revisions of the protocol. This pilot has demonstrated that in the context of the trusting parent–teacher relationships found in ELPs, autism-specific screening of young children and referral to EI can occur at a high rate.

### Limitations

Despite the success of the study, there were limitations.

The nature of working with community-based ELPs has its own innate limitations. Staff turnover and the need to retrain new teaching staff was the largest barrier. To minimize the impact of staff turnover, training focused on changing the environment at the program by training all staff. This process allowed for project continuity when staff members left the ELP. The pilot study’s primary goal was to determine the feasibility of screening in an ELP with a racially/ethnically diverse enrollment. We selected an ELP which was known to enroll a diverse cohort of children. During the first year of the study, race/ethnicity data of individual participants was not collected; in year two, we confirmed that 93% of screened children were Non-Hispanic Black or Hispanic/Latino. In future studies, the collection of race/ethnicity data will be prioritized from study onset.

This study focused on determining feasibility in terms of demonstrating the ability for parent- and teacher-completed autism-specific screening to occur within a single ELP. The study did not evaluate the acceptability of this screening model across stakeholders, implementation burden, fidelity sustainability, cost, scalability, or long-term integration into routine practice; thus, the design of this single-site study cannot evaluate broadly generalized feasibility. These factors should be evaluated in future studies of parent- and teacher-completed autism-specific screening.

A success of this study was the presence of a master’s-level facilitator who developed trusting relationships with families prior to the start of screening. This unique feature of the ELP may not be generalizable to other ELPs completing autism-specific screening.

This study occurred throughout the COVID-19 pandemic. It is notable that the one participant who declined an EI referral did so due to their dislike of remote EI services, which was a unique factor specific to pandemic regulations.

## 5. Conclusions

Our key finding is that autism-specific screening of young children in the ELP setting is feasible for families, teachers, and ELP administrators.

## Figures and Tables

**Table 1:** Fidelity check-in.

Fidelity Check-in Guide	
Site Level	Staff responsible for completing each step of the project protocol
	Number of Children Eligible
	Number of Families Approached
	Number of Families Enrolled in Study
	Number of Families Declining Participation
	Number of Children Screened (Positive and Negative)
	Number of Children Referred to EI
Participant Level	Demographics
	Screening Results
	EI Referral Status (Referred, Not Referred)
	EI Eligibility (Eligible for EI, Not Eligible for EI)
	EI Enrollment Status (Enrolled in EI, Not Enrolled in EI)

## Data Availability

Data is available upon request to the corresponding author. Due to privacy restrictions, data is not available publicly.
